# Acute Hepatitis E in a Young Male

**DOI:** 10.7759/cureus.5414

**Published:** 2019-08-18

**Authors:** Waseem Sarwar Malghani, Waseem Jan, Asim Tameez Ud Din, Ali Raza, Farooq Mohyud Din Chaudhary

**Affiliations:** 1 Gastroenterology, Nishtar Medical University & Hospital, Multan, PAK; 2 Gastroenterlogy, Nishtar Medical University & Hospital, Multan, PAK; 3 Internal Medicine, Rawalpindi Medical University, Rawalpindi, PAK

**Keywords:** hepatitis e, jaundice, acute hepatitis

## Abstract

Hepatitis E (Hep E) is a type of liver disease caused by hepatitis E virus (HEV), which is a single-stranded ribonucleic acid (RNA) virus. This mainly spreads through the intake of contaminated food and water. Here we present a case of a 30-year-old male with complaints of dark-colored urine and yellow discoloration of eyes (jaundice) for the past few days. He also had associated mild abdominal pain, nausea, and loss of appetite. On further inquiry, he pointed out that his drinking water was from an unfiltered source, and had unsatisfactory sanitary conditions at home. On physical examination, he was deeply jaundiced. His laboratory results showed deranged liver function tests (LFTs) and positive serology for HEV. He was managed conservatively and was discharged after improvement in his condition. On follow-up after one month, complete normalization of liver enzymes and symptoms was seen. This case report highlights the significance of better sanitation and personal hygienic habits in the prevention of HEV infection.

## Introduction

Hepatitis E (Hep E) is a form of viral hepatitis which mainly spreads through feco-oral route and is mainly attributed to an unhygienic environment. The prevalence of Hep E is higher in developing countries. It is especially life-threatening for pregnant patients with a maternal mortality rate of 29% [1]. Pakistan being a developing country faces an upsurge of these cases in the regions where there is unavailability of clean drinking water and better sanitation measures. Here we present a case of a 30-year-old male who developed acute hepatitis due to Hep E. This case not only demonstrates the natural history of the disease but also highlights the need of community awareness and education regarding the practice of better cleanliness measures, which can prevent its spread.

## Case presentation

A 30-year-old male from a nearby village area presented to the Gastroenterology department of Nishtar Hospital, Multan in July 2019 with the complaints of yellow discoloration of eyes for three days.

Jaundice was sudden in onset and progressive. The patient reported passing dark-colored urine for the past few days. There were no associated symptoms of itching or clay-colored stools. For the last few days, the patient had symptoms of dull pain of mild intensity in his abdomen associated with loss of appetite and nausea. He reported just one episode of vomiting which did not contain any blood. There was no history of diarrhea, constipation, joint pains, abdominal distension, decreased urinary output, or bleeding from any site of the body. The patient was a married man, school-teacher by profession and nonsmoker and nonaddict. He denied alcohol or any drug use. He had no comorbid illnesses. There was no history of surgery, blood transfusion, dental extraction, or intravenous drug use. He denied illicit sexual behavior. His drinking water was from an unfiltered source and he reported poor sanitary conditions at his home.

On physical examination, he had an average built and height. He was fully conscious and well oriented. There were no flapping tremors. He had a yellow sclera. His abdomen was soft with mild tenderness in the epigastrium and right hypochondrium. There was no visceromegaly or shifting dullness. Rest of the examination was normal.

Upon investigating the patient, he was found to have markedly elevated liver enzymes. His complete laboratory profile is shown in Table 1. His ultrasound of abdomen showed thickening of the gallbladder wall. Liver and spleen were normal. His viral serology revealed the presence of antibodies to Hep E of IgM subtype (immunoglobulin M). Based on his clinical and laboratory evaluation, he was diagnosed as a case of acute hepatitis E virus (HEV) infection.

**Table 1:** Laboratory investigations. HCT, hematocrit; WBC, white blood cells; RBC, red blood cells; MCV, mean corpuscular volume; MCH, mean corpuscular hemoglobin; MCHC, mean corpuscular hemoglobin concentration; ALT, alanine aminotransferase; AST, aspartate aminotransferase; ALP, alkaline phosphatase; GT, glutamyl transferase; A/G, albumin to globulin ratio; PT, prothrombin time; INR, international normalized ratio; BUN, blood urea nitrogen; HBsAg, surface antigen of hepatitis B virus; Anti-HCV, antibody to hepatitis C virus; Anti-HAV IgM, antibodies to hepatitis A virus of immunoglobulin M subtype; Anti-HEV IgM, antibodies to hepatitis E virus of immunoglobulin M subtype.

Hematology report		Blood chemistry	
Hemoglobin	14.2 g/dL	Total bilirubin	10.6 mg/dL
RBC count	4.9 x 10^12^ /L	Bilirubin conjugated	6.4 mg/dL
HCT	42%	Bilirubin unconjugated	4.1 mg/dL
MCV	86 fL	ALT	1783 U/L
MCH	29 pg	AST	1591 U/L
MCHC	33 g/dL	ALP	183 U/L
Platelets count	185 x 10^9^/L	Gamma GT	86 U/L
WBC	10.3 x 10^9^/L	Total proteins	6.7 g/dL
Neutrophils	74%	Albumin	3.5 g/dL
Lymphocytes	14%	Globulins	3.2 g/dL
Monocytes	09%	A/G ratio	1.1
Eosinophils	03%		
Serum electrolytes		Coagulation tests	
Sodium	137 mmol/L	PT	16 sec
Potassium	3.8 mmol/L	INR	1.5
Chloride	106 mmol/L	Renal function test	
Bicarbonate	22 mmol/L	Serum creatinine	0.7 mg/dL
Viral Serology		Serum urea	21 mg/dL
Anti-HEV IgM	Reactive	BUN	10 mg/dL
Anti-HAV IgM	Nonreactive	Chemical pathology	
HBsAg	Nonreactive	Serum amylase	55 U/L
Anti HCV	Nonreactive	Serum lipase	14 U/L

The patient was hospitalized in the general ward for a few days as he was unable to tolerate oral intake. He was given intravenous (IV) dextrose containing fluids, IV proton pump inhibitors (PPI), IV antiemetics, and lactulose syrup. Gradually his nausea improved, and he was able to tolerate liquids and later on a soft diet. He was encouraged to take fresh juices and milkshakes. His liver enzymes started returning to normal levels. He was discharged after a few days. Follow-up after two weeks and then after one month showed complete normalization of liver enzymes and his symptoms. He was advised for frequent hand washing especially before eating or handling food and use of filtered water source for drinking purposes. 

## Discussion

Hepatitis E virus is a spherical, single-stranded RNA virus. The two common genotypes identified in humans are HEV1 and HEV2. The pathogenesis of Hep E depends upon multiple factors including genetic predisposition and immunological responses to HEV. In multiple in vitro studies, interferon-alpha (IFN-α) is found to play a vital role in the manifestations of Hep E symptoms by inhibiting certain antiviral proteins [2]. The transmission of HEV is mainly due to unsanitary conditions. Our patient used to drink water from an unfiltered source and had poor sanitary conditions at his home. The most common symptom in acute infection is jaundice, which was also seen in our patient. Other manifestations include constitutional symptoms like fever, fatigue, joint pain, and gastrointestinal features [3].

The diagnosis of acute Hep E is made by confirming the presence of antibodies against HEV (IgM subtype). For some patients who are immunocompromised, nucleic acid amplification tests (NATS) can be useful to detect HEV ribonucleic acid (RNA) if serology is negative. Our patient was diagnosed on the basis of positive anti-HEV IgM antibodies [4]. The prognosis of Hep E depends on the age and sex of the patient. Generally younger age, female sex, and pregnancy are considered as bad prognostic signs as these patients can develop acute liver failure. Our patient was a young male and was successfully managed with no long-term sequelae [5].

The management of Hep E is mainly supportive. Majority of the cases are self-limiting, as seen in our case. The use of certain therapies like ribavirin and steroids in patients who had a severe infection and acute liver failure respectively has been reported in the literature but more studies are required to monitor their long-term effects [4]. The most important aspect of its management is prevention. Although a vaccine has emerged for protection against HEV infection, it is not available in all countries. To prevent outbreaks especially in developing countries, people should be properly educated to follow simple hygienic principles related to hand washing, food, and water intake [6].

## Conclusions

Hepatitis E is a viral illness which commonly transmits by the feco-oral route. It is mainly attributed to the unhygienic conditions. The management is mainly supportive in most of the cases. In certain high-risk conditions like pregnancy, there could be the development of acute liver failure which should be treated as a medical emergency. Our patient developed acute HEV infection and was successfully managed.
